# Correlated responses to selection across diverse environments during experimental evolution of *Tetrahymena thermophila*

**DOI:** 10.1002/ece3.11395

**Published:** 2024-07-23

**Authors:** Jason Tarkington, Rebecca A. Zufall

**Affiliations:** ^1^ Department of Biology and Biochemistry University of Houston Houston Texas USA; ^2^ Department of Genetics Stanford University Stanford California USA

**Keywords:** Correlated Responses, Experimental Evolution, Tetrahymena

## Abstract

Correlated responses to selection have long been observed and studied; however, it remains unclear when they will arise, and in what direction. To contribute to a growing understanding of correlated responses to selection, we used experimental evolution of the ciliate *Tetrahymena thermophila* to study direct and correlated responses in a variety of different environmental conditions. One experiment focused on adaptation to two different temperatures and the correlated responses across temperatures. Another experiment used inhibitory concentrations of a variety of compounds to test direct and correlated responses to selection. We found that all populations adapted to the environments in which they evolved. We also found many cases of correlated evolution across environments; few conditions resulted in trade‐offs and many resulted in a positive correlated response. Surprisingly, in many instances, the correlated response was of a larger magnitude than the direct response. We find that ancestral fitness predicts the extent of adaptation, consistent with diminishing returns epistasis. Unexpectedly, we also find that this pattern of diminishing returns holds across environments regardless of the environment in which evolution occurs. We also found that the correlated response is asymmetric across environments, that is, the fitness of a population evolved in one environment and assayed in a second was inversely related to the fitness of a population evolved in the second environment and assayed in the first. These results support the notion that positive correlated responses to selection across environments are frequent, and worth further study.

## Graphical abstract

Populations of *Tetrahymena thermophila* were evolved in many different environments, and the evolutionary response of each population was measured across the set of all environments. Correlated responses tended to be positive and were often larger than the direct responses.

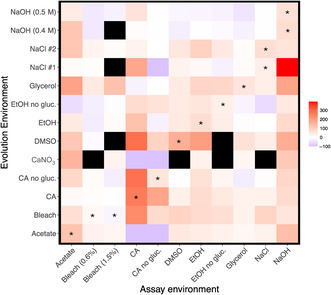

## 1 INTRODUCTION

Populations face continuously changing environments that impose varying selective pressures. Adaptation to these novel environments usually results in an increase in fitness in the new environment but may also be accompanied by correlated responses to selection, that is, evolutionary changes in traits not directly under selection. Such correlated responses occur because of underlying genetic correlations, which may be caused by pleiotropy or linkage disequilibrium (Garland et al., 2022; Lande, 1982). Correlated responses to selection are of particular interest, in part, because they have the potential to constrain or facilitate future adaptation in populations (Acerenza, 2016) and contribute to the evolution of diversity (Burmeister et al., 2020).

Correlated responses to selection have long been observed and studied (e.g., Darwin, 1859). Often, these responses result in trade‐offs, whereby increased fitness in one environment or trait leads to decreased fitness in another environment or trait (Stearns, 1989). Trade‐offs have been found in a wide variety of traits, across taxonomic groups (e.g., Agudelo‐Romero et al., 2008; Gompert & Messina, 2016), and have been the subject of many theoretical studies (e.g., Martin & Lenormand, 2015; Shoval et al., 2012). Indeed, trade‐offs are so common that it is often assumed that evolution in response to selection on one trait or in one environment will be associated with trade‐offs that decrease fitness in other traits or environments (Agrawal et al., 2010; Van den Bergh et al., 2018). However, trade‐offs are not always found; it is also possible that selection on one trait or in one environment increases fitness in another, resulting in a positive correlated response. Positive correlated responses have been found in a variety of contexts, usually unexpectedly (e.g., Magalhães et al., 2009; Nidelet & Kaltz, 2007; Olazcuaga et al., 2021). Despite such intense scrutiny, it remains unclear under precisely what conditions various types of correlated responses will evolve (Bono et al., 2017; Jasmin & Zeyl, 2013; Van den Bergh et al., 2018).

In this study, we test for correlated responses to selection by experimentally evolving populations in one environment and measuring their evolved fitness across a variety of environments. Experiments using similar approaches have found mixed results. Sometimes, trade‐offs are found, for example, between growth in light versus dark in *Chlamydomonas* (Bell & Reboud, 1997) and across temperatures following adaptation at 37°C in *Escherichia coli* (Cooper et al., 2001). Sometimes, however, a variety of types of correlated responses are found even within a single experiment. Bennett and Lenski (2007) found that trade‐offs across temperature regimes in *E. coli* were general; however, many evolved lineages showed no evidence of trade‐offs, and one showed a positive correlated response. Evolving *E. coli* in acidic versus alkaline environments resulted in trade‐offs when acidic‐adapted lines are grown in alkaline environments, but not the reverse (Hughes et al., 2007). In yeast, evolution under resource limitation leads to trade‐offs in high resource environments. However, no such trade‐offs were found when grown in other resource‐limited environments; rather, increased fitness was found across resource‐limited environments showing evidence of positive correlated responses (Wenger et al., 2011).

Here, we use the microbial eukaryote *Tetrahymena thermophila* to further study correlated responses to selection. *Tetrahymena thermophila* are ciliates, endemic to the northeastern United States, and an important part of the microbial food loop in freshwater ponds and streams (Doerder, 2019; Weisse, 2017; Zufall et al., 2013). Microbes are particularly susceptible to environmental perturbations and selective pressures due to direct contact with their environment and high surface area to volume ratio. In particular, ciliates, like many aquatic protists, respond quickly to external stimuli due to the lack of a cell wall (Esteban & Fenchel, 2021; Gomiero et al., 2012) and propensity to ingest nano‐ and micro‐scale particles (Frankel, 2000; Mortimer et al., 2010). Because of this, ciliates are often used for toxicological assessment (e.g., Bogaerts et al., 2001; Bonnet et al., 2003, 2005, 2008; Kurvet et al., 2017; Nilsson, 1989; Sauvant et al., 1999; Schultz et al., 2005). Their short generation time and large population size also give them the ability to quickly adapt to novel conditions. Thus, ciliates make a valuable system in which to study correlated responses to selection across diverse environments.

We use experimental evolution of *T. thermophila* to test for correlated responses to selection using two different selective scenarios. In the first experiment, we evolve populations in “benign” environments. Populations are selected for growth at two different temperatures, where growth rates differ, but growth is not inhibited and cells show no evidence of stress. Evolution in response to temperature, including direct and correlated responses, has been studied in a variety of contexts (Angilletta et al., 2010; Buckley & Kingsolver, 2021; Malusare et al., 2023), and a meta‐analysis of experimental evolution and artificial selection studies found that adaptation to higher temperatures often results in trade‐offs with fitness at lower temperatures (Malusare et al., 2023). In the second experiment, evolution occurs in a variety of stressful environments that contain compounds that inhibit growth. In this experiment, rather than studying correlated responses across specific environments, we ask more generally how selection in one stressful environment affects performance in other stressful environments. In both experiments, we measure the adaptive response in the evolution environment and the correlated response in the alternate environments to assess whether correlated responses are common across environments, and if so, whether the direction of the response tends to be positive or negative, that is, trade‐offs. In both experiments, we find little evidence for trade‐offs, but many instances of positive correlated evolutionary responses across environments. Our results add to the growing body of literature that examines under what conditions varying types of correlated responses to selection are likely to be found.

## 2 METHODS

### 2.1 Overview

We performed two independent experiments to assess how evolution in one environment affects fitness in an alternate environment. In the first experiment, we used three different genotypes of *T. thermophila* to start replicate populations that were evolved at two different temperatures. Fitness was assayed for all evolved populations at both temperatures. In the second experiment, we evolved one population of a single genotype in many different environments containing various compounds that inhibit growth. Following the evolution, fitness of each evolved population was assayed across these environments.

### 2.2 Temperature adaptation

For this experiment, we used three genotypes of *T. thermophila*: two natural isolates, strain 19617‐1, which we call A (*Tetrahymena* Stock Center ID SD03089) and strain 19625‐2, which we call B, and an offspring of a cross between these two strains, A × B. The cross and experimental evolution procedures are described in Tarkington and Zufall (2021). Briefly, four replicates of each genotype were allowed to evolve in nutrient‐rich medium (SSP; Gorovsky et al., 1975) for 4000 generations at either 24 or 37°C in nutrient‐rich medium. This resulted in 24 evolving populations (3 genotypes × 4 replicates × 2 temperature treatments). Cells were transferred to fresh medium daily. Growth rate was measured as a component of fitness by growing cells in a microplate reader. Populations were randomized across 96‐well plates; OD_650_ readings were taken every 5 min over the course of the growth curve; and an R script was used to estimate the maximum slope of log‐transformed growth curves (Long et al., 2013). The growth rate of all populations was measured at both temperatures. Growth rate measurements were binned across generations 3875–4125, and the mean growth rate was calculated for that time period. Relative change in growth rate was calculated for each population as the difference between the ancestor and evolved growth rate divided by the ancestral growth rate × 100 to indicate the percent change from the ancestor. The ancestral growth rate was estimated from growth curves calculated over the first 50 generations. Growth rate data were analyzed using a standard least squares fit ANOVA with population treated as a random variable to test the effect of assay temperature, evolution temperature, genotype, and their interactions on changes in growth rate.

### 2.3 Novel environment adaptation

For this experiment, all populations were started from the laboratory strain SB210‐E (*Tetrahymena* stock center ID SD01539). Each population was allowed to evolve in SSP containing inhibitory levels of various organic or inorganic compounds (Table 1). In some cases, the concentration was lower at the start of the experiment and was gradually increased as the population began to grow faster and survive in concentrations that were previously lethal (see starting concentration vs. final concentration in Table 1).

**TABLE 1:** Conditions of media for novel environment adaptation.

Compound	Starting concentration	Final concentration	Assay concentration
Glycerol	2%	5%	5%
Ethanol	2%	3%	1.5%
Ethanol (no glucose)	2%	3%	1.5%
Bleach	1.5%	1.5%	0.6% and 1.5%
Citric acid	0.12%	0.18%	0.162%
Citric acid (no glucose)	0.12%	0.18%	0.162%
NaOH^a^	0.02 M	0.04 and 0.05 M	0.025 M
Acetate	15 g/L	15 g/L	15 g/L
CaNO_3_	15 g/L	15 g/L	N/A
NaCl^b^	15 g/L	25 g/L	15 g/L
DMSO	2%	4%	2.5%

^a^ Two populations were grown in NaOH and finished the experiment at two different concentrations.

^b^ Two replicate populations were grown in NaCl.

Thirteen populations of a single genotype of *T. thermophila* were evolved for at least 2000 generations in 12 different culture conditions, each containing different compounds that inhibited the growth of ancestral cultures. Due to the slow rate of cell division under these conditions, this experimental evolution lasted between 3 and 5 years. All populations were then assayed for growth rate and carrying capacity in 11 different environments containing these compounds to estimate evolved fitness in the environment in which they evolved (direct responses) and in alternate environments (correlated responses).

Populations were maintained by serial dilution in 10 mL of cultures. Dilution factors were adjusted so that daily bottlenecks never fell below ~20,000 cells and effective population size was maintained at ~100,000 cells. Slower growing cultures experienced a larger bottleneck size, but did not grow to as high of a density as faster growing populations. After at least 2000 generations of evolution, we measured the maximum population growth rate, as described above, and carrying capacity, as maximum OD, of each evolved population and the ancestor (thawed from stock) in each of the unique environments, except for CaNO_3_, due to the formation of precipitate in this medium. At least four growth curves per population were obtained for each assay environment. For some measurements, the concentration of the inhibitory compound was reduced to allow for growth of the populations (see assay concentration in Table 1). Two concentrations of bleach were used in assays. Relative change in fitness components (either growth rate or carrying capacity) was calculated as the difference between the ancestor and evolved population divided by the ancestor fitness. Ancestral growth curves were collected on the same plates as evolved populations. Standard least squares fit ANOVAs were used to test for differences among populations evolved in different environments. Since there were no replicate populations within a single condition, no tests were performed between specific environments.

## 3 RESULTS

### 3.1 Temperature adaptation

**FIGURE 1:**
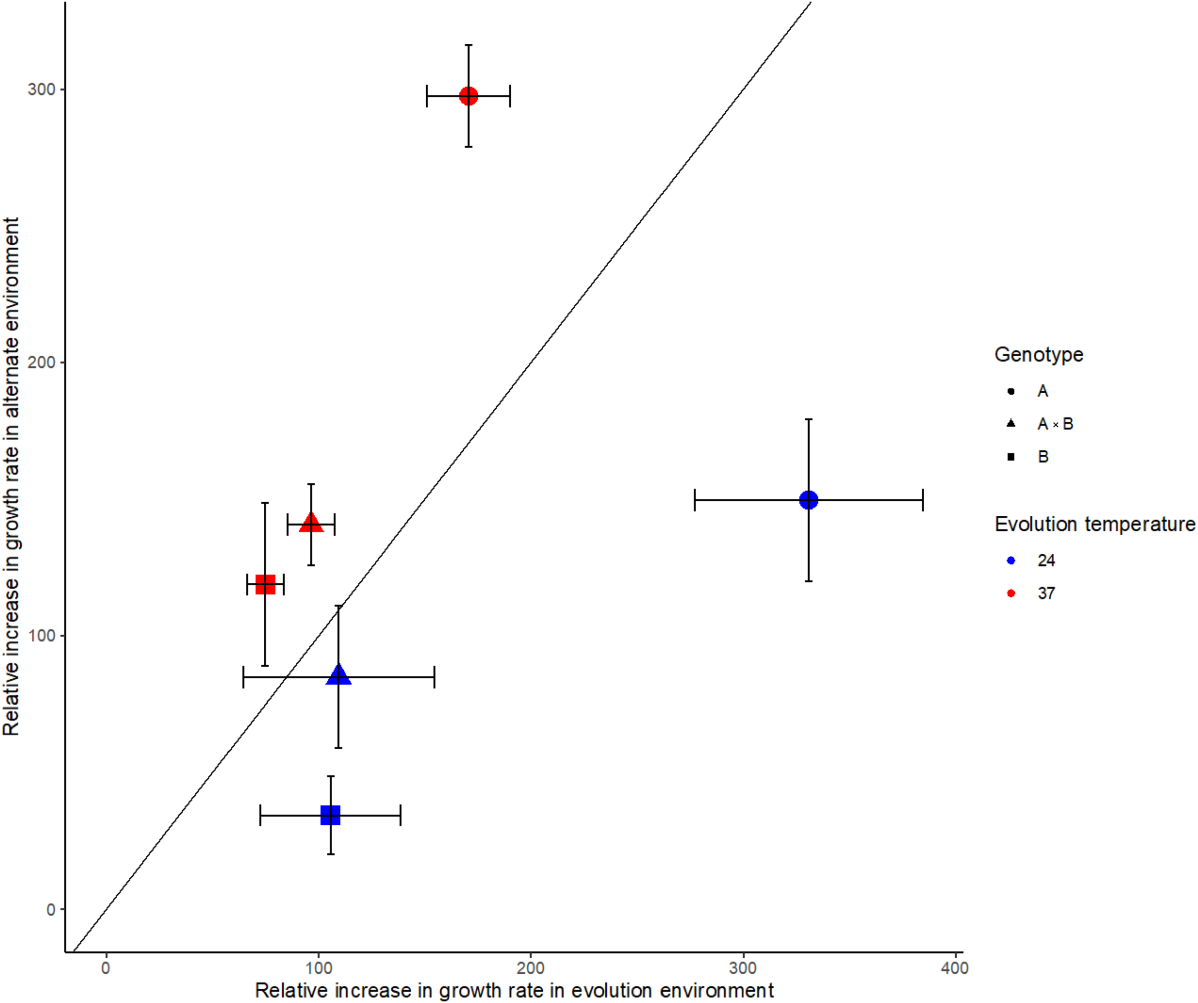
Relative increase in growth rate in evolution environments versus alternate environments. Positive values indicate that growth rates are higher than those of the ancestor. The *x* = *y* line indicates where the fitness increase is the same in both environments. Points above this line indicate a higher fitness gain in the alternate environment. Populations evolved at 24°C are shown in blue; 37°C in red. Genotypes are represented by different shapes. Error bars are standard deviations among four replicate populations. Relative increase is shown as a percent.

Replicate populations of three genotypes of *T. thermophila* were evolved at 24 or 37°C for 4000 generations. The growth rate of each population was measured in both the temperature in which it evolved and the alternate temperature. All populations evolved higher growth rates in both their evolution and alternate temperatures indicating no evidence of evolutionary trade‐offs, but rather a positive correlated response to evolution despite differences in temperature regimes (Figure 1). The 24°C evolved populations had a higher direct response, that is, their growth rate increased more at 24°C than at 37°C (blue points below the *x* = *y* line in Figure 1). In contrast, the 37°C evolved populations had a greater correlated response, with their growth rate increasing more in the temperature that they did not evolve in (red points above the *x* = *y* line in Figure 1). In fact, on average, the 37°C evolved populations had greater relative increases at 24°C than populations evolved at 24°C (191% vs. 187%). A similar observation was made in flies where evolution at 24°C resulted in greater tolerance at 16°C compared to when evolution occurred at 16°C (Condon et al., 2015).

An ANOVA was used to determine which factors contribute to the differences in growth rate between populations. Assay temperature, but not evolution temperature, significantly affected the relative increase in growth rate, as did genotype and the interaction between assay temperature and genotype and assay temperature and evolution temperature (ANOVA: *F*_AssayT_ (12668) = 1147.7, *p* < .0001; *F*_EvoT_ (12668) = 2.89, *p* = .1064; *F*_genotype_ (22667) = 120.77, *p* < .0001; *F*_AssayTxEvoT_ (12668) = 10.69, *p* = .0011; *F*_AssayTxgenotype_ (22667) = 209.84, *p* < .0001; *F*_EvoTxgenotype_ (22667) = 1.13, *p* = .3453). In particular, the genotype with the lowest initial growth rate (genotype A, shown as circles in Figure 1) showed significantly larger increases than the other genotypes in both the evolution and alternate environments (Table 2); and the assay temperature with the lowest initial growth rate (24°C) showed the largest increases following evolution. This is consistent with diminishing returns epistasis (Chou et al., 2011).

**TABLE 2:** Comparison of increases in growth rate between genotypes at each evolution and assay temperature.

Evolution	Assay	Genotypes compared (*p*‐value)^a^		
		A vs. B	A vs. A × B	B vs. A × B
24°C	24°C	**<.0001**	**<.0001**	.905
24°C	37°C	**<.0001**	**.0044**	**.016**
37°C	24°C	**<.0001**	**<.0001**	.196
37°C	37°C	**<.0001**	**<.0001**	.0558

^a^ *p*‐values from Student's *t*‐tests; bold values indicate significant differences between genotypes; *T* = 2.26.

### 3.2 Novel environment adaptation

**FIGURE 2:**
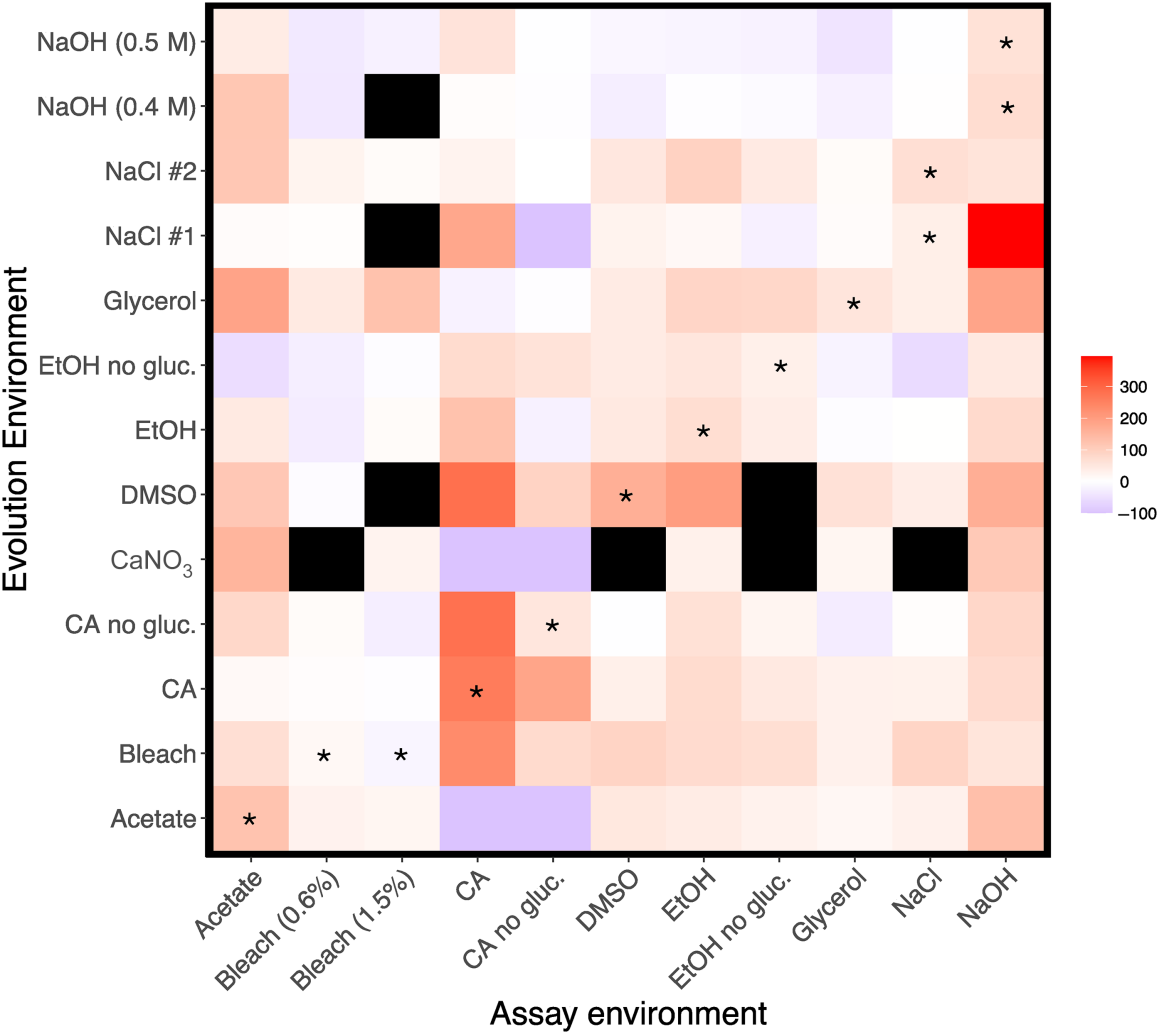
Heat map showing the relative percent change in growth rate in 11 environmental assay conditions of the 13 populations evolved in different environments. Each square shows the mean fitness change in an assay environment for a population evolved in a given environment (Table 1). Stars indicate the same evolution and assay environments. Gray squares are missing data. NaCl #1 and #2 indicate duplicate populations evolved in NaCl. Citric acid is abbreviated CA; ethanol as EtOH. Relative change is shown as a percent.

Single populations of *T. thermophila* were evolved in several stressful environments for at least 2000 generations. Populations evolved in some of these environments showed increases in growth rate across most environments (shown as darker red squares in a row in Figure 2, e.g., glycerol, DMSO, CA, NaCl #2; proportion of positive correlated responses can be seen in Table S1), while others showed little increase in growth rate in any environment (rows with light red and blue squares, e.g., populations evolved in EtOH with no glucose and NaOH; Table S1). Likewise, some environments tend to result in large increases in growth rate regardless of the evolution environment (columns with more red in Figure 2, e.g., acetate and NaOH), while in other environments growth rates show little increases for any evolved populations (columns with light red and blue in Figure 2, e.g. EtOH with no glucose and glycerol). An ANOVA was used to assess whether populations evolved in different environments have different growth rates and carrying capacities. Both evolution environment and assay environment significantly affect relative increase in growth rate (*F*_Evo_ (12,133) = 4.767, *p* < .0001; *F*_Assay_ (9136) = 3.063, *p* < .0006) and carrying capacity (*F*_Evo_ (12,132) = 4.734, *p* < .0001; *F*_Assay_ (9135) = 5.960, *p* < .0001). Note that the effect of the evolution environment is not replicated so that while we can tell if evolved genotypes are different from one another these differences may not be representative of the mean responses if populations were replicated within each evolution condition.

**FIGURE 3:**
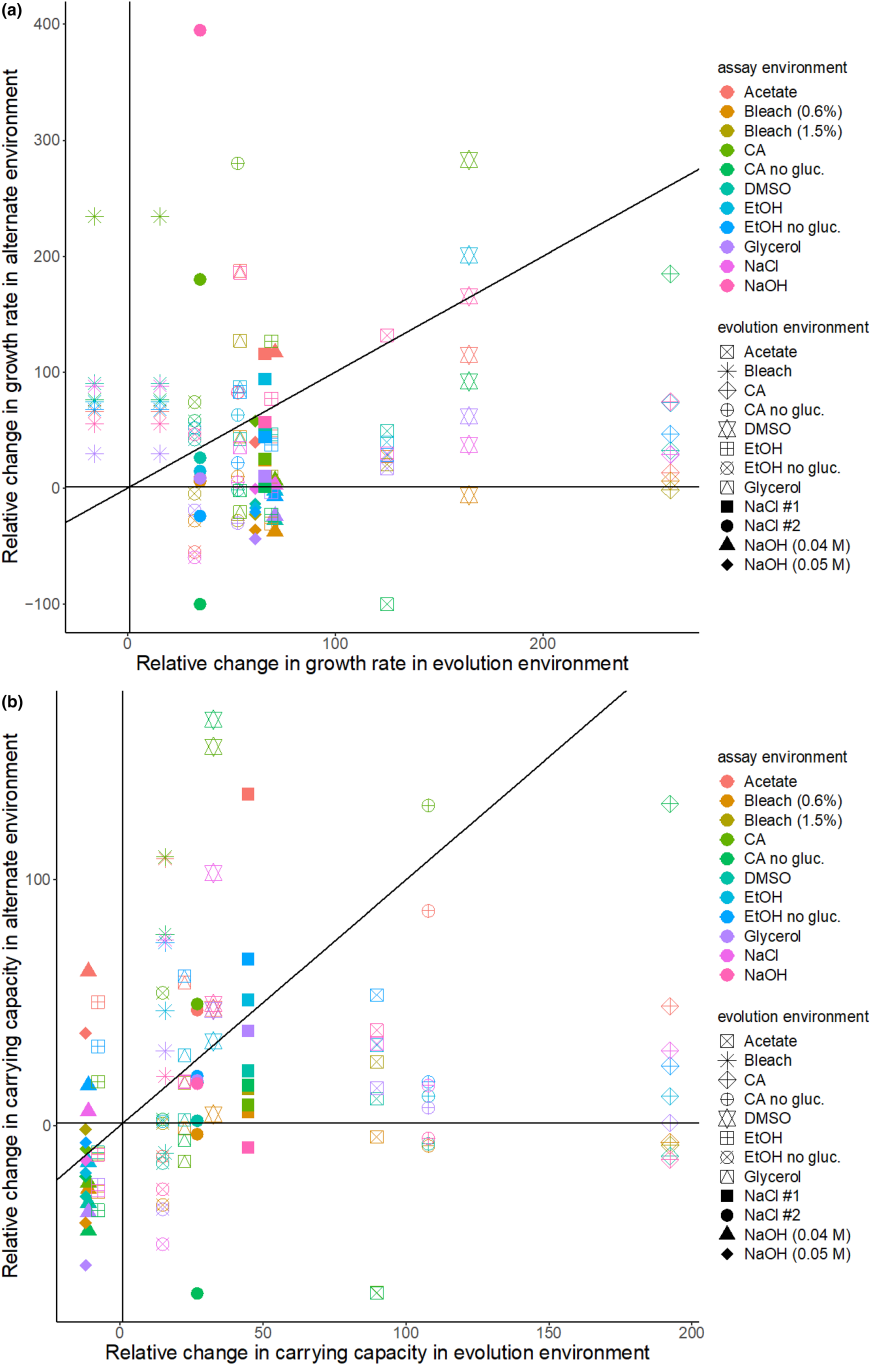
Relationship between relative change in fitness in evolution environment versus alternate environments. Each point is the growth rate (a) or carrying capacity (b) of an evolved population in its evolution environment (*x*‐axis) and in one of the alternate environments (*y*‐axis). The shape of the point indicates the evolution environment and the color indicates the assay environment. Lines are shown at *x* = 0, *y* = 0, and *x* = *y* to identify trade‐offs or patterns of correlated response. Note that the bleach evolved strain is shown in its evolution environment for both concentrations of bleach that were assayed (0.6% and 1.5%). Relative change is shown as a percent.

To determine the direction of the effect of evolution environment on fitness in alternate environments, that is, trade‐offs versus positive correlated responses, we plotted the relative change in fitness in the evolution environment against the relative change in alternate environments (Figure 3). We find several instances of trade‐offs (for growth rate 35/135 points below zero on the *y*‐axis and greater than zero on the *x*‐axis, and 21/134 for carrying capacity; Figure 3), but most populations show positive correlated increases in fitness across environments (positive values in Figure 3). A ranked correlation of the data in Figure 3 (not shown) is not significant for growth rate (spearman; *r* = −.12, *p =* .19) but it is for carrying capacity (spearman; *r* = .35, *p =* .00015), supporting an overall positive correlated response to evolution across environments in carrying capacity. Because we lack replicates within an environment, we cannot address whether a particular environment is more or less likely to result in positive correlated responses to selection.

Our expectation was that populations would increase in fitness more in their evolution environment than in an alternate environment, that is, direct responses would be greater than correlated responses, following a pattern of local adaptation (Whitlock, 1996). Bono et al. (2017) show that this should be the case particularly during selection in homogeneous environments, as in our experiments. However, we observe many instances of larger increases in fitness in alternate environments than evolution environments (points falling above the *x* = *y* line in Figure 3; 43/124 for growth rate and 35/115 for carrying capacity). In addition, the three largest increases in relative growth rate were found in alternate environments rather than the environment where the population evolved (Figure 3a, evolved in NaCl, assayed in NaOH; evolved in CA no glucose, assayed in CA; evolved in DMSO, assayed in CA).

**FIGURE 4:**
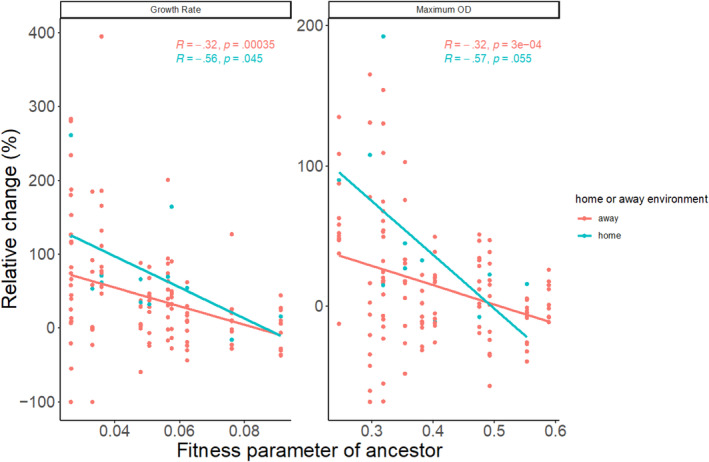
Relationship between ancestral fitness parameters (growth rate or maximum OD_650_) and change in fitness following evolution. Each point shows the fitness of the ancestor in one of the 11 environments (*x*‐axis) versus the relative change in fitness in that environment following evolution either in that environment (“home”; blue points) or a different environment (“away”; red points). A linear regression was fit for the direct (blue) and correlated (red) responses. Regression coefficients and *p*‐values are shown for each analysis.

To further explore what factors contribute to the observed changes in fitness across environments, we asked whether ancestral fitness in a given environment affects the resulting increase in fitness following evolution, as would be predicted by diminishing returns epistasis (Persson et al., 2022; Wünsche et al., 2017). We plotted the fitness of the ancestor in an environment against the relative change in fitness in that environment for all evolved populations. We found that lower ancestral fitness leads to larger increases in fitness for both fitness parameters measured (growth rate and carrying capacity) across all populations (Figure 4). This result holds for both the direct response to selection, that is, populations evolved and assayed in the same environment (“home,” blue lines in Figure 4; significant for growth rate and marginally significant for maximum OD), and for correlated responses to selection, that is, populations assayed in a different environment than where they evolved (“away,” red lines in Figure 4). Note that for Maximum OD when the leftmost points, that is, those with the smallest values, are removed the significance for the direct responses in the home environment increases to *p* = .0955 while the away response remains significant (*p* = .0346).

**FIGURE 5:**
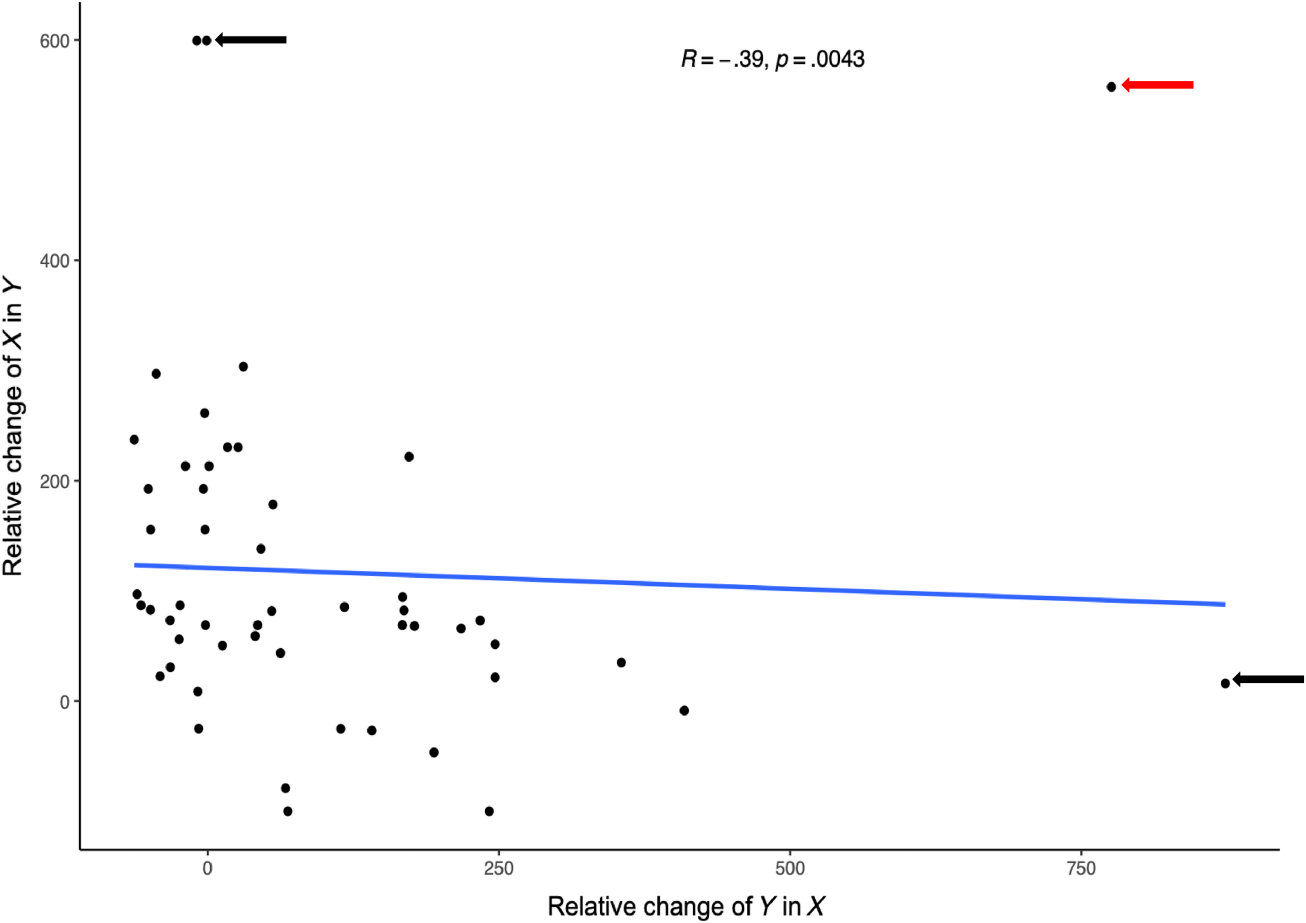
Asymmetry of the correlated response to selection across environments. Each point shows one of 53 unique combinations of two environments. The *y*‐axis shows the relative fitness change of a population evolved in environment *X* and assayed in environment *Y*, and the *x*‐axis shows the fitness change of a population evolved in *Y* and assayed in *X*. The fitness metric shown is *r*‐max × maximum OD. Spearman correlation coefficient is shown. The arrows indicate points with a Mahalanobis distance of greater than 2.93 (α = .01). The Red arrow shows the combination of populations for citric acid and citric acid with no glucose and is the most extreme outlier. Relative change is shown as a percent.

Finally, we ask whether the fitness change observed when a population evolves in environment *X* and is assayed in environment *Y* is correlated with the fitness change for a population evolved in environment *Y* and assayed in environment *X*. We found a significant negative correlation between these measures for the composite fitness trait growth rate × maximum OD (Spearman correlation, *R* = −.39, *p* = .0043; Figure 5) indicating an asymmetric response to selection across assay environments. (This correlation was not significant when growth rate or maximum OD was considered alone (Spearman correlation, growth rate: *R* = −.26, *p* = .0581; maximum OD: *R* = .24, *p* = .078).) This means that if a population evolved in environment *X* has a large increase in fitness in environment *Y*, then a population evolved in environment *Y* is likely to have a smaller change when assayed in environment *X*. An outlier analysis revealed 4 points (indicated by arrows in Figure 5) with Mahalanobis distances (Mahalanobis, 2018) of greater than 2.93 (α = .01). When these data points are removed the correlation was significant for the composite metric (growth rate × maximum OD) and growth rate but not for maximum OD alone (Spearman correlation, growth rate: *R* = −.2961, *p* = .0388; maximum OD: *R* = −.2572, *p* = .0745; composite metric: *R* = −.4339, *p* = .0018). The most extreme outlier—the point indicated by the red arrow in Figure 5 is for the environments citric acid and citric acid with no glucose. Considering the similarity of these environments, it is perhaps not surprising that their fitness increases are positively correlated. With only this outlier removed, the negative correlation is significant for all fitness components measured (Spearman correlation, growth rate: *R* = −.32, *p* = .0211; maximum OD: *R* = −.31, *p* = .0252; composite metric: *R* = −.46, *p* = .0006).

## 4 DISCUSSION

To better understand the role of trade‐offs and correlated responses during adaptation, we evolved populations under a variety of conditions and measured their change in fitness in both the environment in which they evolved and alternate environments. In one experiment, we studied evolution under two different “benign” temperature regimes, both of which allowed rapid growth of *Tetrahymena* populations. In the second experiment, we studied evolution in “harsh” environments, where ancestral populations struggled to grow. In both experiments, all populations show a direct response to selection as expected, that is, all populations increase in at least some component of fitness in the environment in which they evolved. However, in neither experiment, do we find strong evidence for pervasive trade‐offs. There are some cases of populations that exhibit trade‐offs in the novel environment experiment, but none in the temperature experiment. Instead, we find frequent positive correlated responses to selection where evolution in one environment leads to increased fitness not only in that environment, but also in other environments. Surprisingly, in both experiments, we find many cases of correlated responses to selection that are larger than the direct response.

Often, it is assumed that there will be trade‐offs in fitness across environments (Agrawal et al., 2010); however, our results are broadly in agreement with several other experimental evolution studies that show that positive correlated responses to selection may be abundant (McNamara & Simmons, 2017; Nidelet & Kaltz, 2007; Olazcuaga et al., 2021; Van den Bergh et al., 2018). In our temperature experiment, all populations showed a positive correlated response across temperatures in contrast to results from *E. coli* which tended to evolve reduced fitness at a 40°C when evolution occurred at 20°C (Bennett & Lenski, 2007). This may be due to general adaptation to the laboratory environment. Since populations were started with natural isolates that had not been grown in laboratory for long periods of time, it is possible that the observed fitness gains were due to mutations that generally increased growth under our laboratory culture conditions. While all populations increased in fitness at the alternate temperature the correlated response was greater when evolution occurred at the hotter temperature, a finding that is broadly consistent with the previous results (Bennett & Lenski, 2007; Condon et al., 2015). Exploration of how the full thermal performance curves evolve is necessary to further elucidate how evolution at one temperature affects performance at other temperatures (Malusare et al., 2023).

The novel environments experiment also resulted in many cases of positive correlated response; however, in this experiment, we used a strain that has been cultivated under laboratory conditions for decades (Eisen et al., 2006). Thus, we do not expect to find large changes in fitness due to selection from the laboratory environment. In addition, in this experiment, populations experienced highly diverse environments that imposed strong selection. Therefore, we expected to find far fewer positive correlated responses since we had no reason to expect that evolution to survive in the presence of one inhibitory compound should increase the ability to survive in another. However, we found that this was not the case. There are at least two possible explanations for this observation. First, it may be that we again are selecting for increased growth under general lab conditions, which would result in increased fitness under many of the novel environments. Alternatively, it may be that the mechanisms that evolve for dealing with inhibitory compounds are similar, and therefore pleiotropic regardless of the exact nature of the compound, which would result in increased fitness across environments. Previous studies have found that shared molecular mechanisms can explain some positive correlated responses to selection (e.g., Reyes et al., 2013; Rodriguez‐Verdugo et al., 2013). Our experimental design does not allow us to determine the strength of the correlation across specific environments, but if shared mechanisms, such as transport channels, for dealing with inhibitory compounds were responsible for the correlated response, we might predict that compounds that are more similar to each other should result in strong correlated responses. However, Rodriguez‐Verdugo et al. (2013) found a shared mechanism underlying the evolution of antibiotic resistance as a correlated response to high‐temperature selection. Thus, the expected pattern may not be so clear, and molecular characterization and analyses of mutational effects on fitness in different environments may be required to determine the underlying causes of positively correlated responses across environments.

Unexpectedly, we found that in many cases, the positive correlated responses to selection were larger in magnitude than the direct response (points above the diagonal line in Figure 3) and the direct response was greatest in only 1/13 conditions for growth rate (Figure 3a; acetate) and 2/13 conditions for carrying capacity (Figure 3b; acetate and citric acid). In the temperature experiment, this phenomenon was only observed for the populations evolved under the higher temperature (Figure 1). In the novel environments experiment, there was no clear pattern as to which populations had a larger correlated response (Figure 3). Further, in some cases, the largest increase in fitness for a particular assay environment is seen in a population evolved in a different environment, for example, in the NaOH environment an NaCl evolved population had larger increases in growth rate than populations that were evolved in NaOH. These counterintuitive results may be explained by a toolbox model for the evolution of trade‐offs described by Tikhonov et al. (2020). However, differences in the mutation rate between environments could also be responsible (Tikhonov et al., 2020). It is also possible that this outcome is idiosyncratic and underlain by different mechanisms depending on the particular genotypes and environments.

In the novel environments experiment, we found that ancestral fitness parameters are a good predictor of the extent of evolutionary change. This pattern, likely due to diminishing returns epistasis, has been well‐documented in a diversity of systems (Couce & Tenaillon, 2015; MacLean et al., 2010; Wiser et al., 2013). Surprisingly, we found that this pattern was consistent regardless of the environment in which evolution occurs, that is, even in the “away” environments, the amount of evolutionary change could be predicted by the ancestral fitness in that environment. This novel finding is consistent with Wei and Zhang's (2019) demonstration that environment quality can predict the fitness effects of beneficial mutations. It is also consistent with our finding of many correlated responses being greater than direct responses because for many environments in our experiment there are other environments with a lower initial growth rate (where the same mutations could have greater effects). Both of these findings are also consistent with our observation that assay temperature but not evolution temperature effect the relative increase in growth rate.

The novel environments experiment also revealed an asymmetric response to selection, where the fitness of a population evolved in one environment and assayed in a second was inversely related to the fitness of a population evolved in the second environment and assayed in the first. A similar type of asymmetry was also found in a study of a resistance—development‐time trade‐off, and provides evidence that the traits under selection are likely underlain by multiple genes (Bartlett et al., 2020). Future studies to identify the beneficial mutations in these populations will clarify whether there exist differing targets of selection in different environments that could explain the asymmetry.

Our results here add to the growing body of evidence that positive correlated responses to selection are frequent, at least in experimental evolution settings. Continued research into the genetic mechanisms underlying these responses will shed light on the roles of various evolutionary mechanisms driving the correlation. We advocate for continued research in this area utilizing diverse taxa, which are likely to provide valuable insight that may not be readily uncovered with traditional model systems.

## AUTHOR CONTRIBUTIONS

**Jason Tarkington:** Conceptualization (lead); data curation (lead); formal analysis (lead). **Rebecca A. Zufall:** Writing – review and editing (equal).

## FUNDING INFORMATION

NSF grant # 1911449.

## CONFLICT OF INTEREST STATEMENT

The authors have no conflict of interests to report.

### OPEN RESEARCH BADGES

This article has earned an Open Data badge for making publicly available the digitally‐shareable data necessary to reproduce the reported results. The data is available at https://doi.org/10.5061/dryad.vx0k6djzb.

## Supporting information


Data S1:

## Data Availability

All data will be posted to dryad: https://doi.org/10.5061/dryad.vx0k6djzb. The data has also been uploaded as a Supporting Information.
