# Supplementary material for: Correlated responses to selection across diverse environments during experimental evolution of Tetrahymena thermophila
Source: Ecol Evol. 2024 Jul 23;14(7):e11395. doi: 10.1002/ece3.11395 (PMC11264346; doi:10.1002/ece3.11395)
Supplement: Supplementary file 1 — Data S1: [file ECE3-14-e11395-s001.zip › ms3 supplemental table 041624.docx]

| Evolution Environment | Positive correlated response | Negative Correlated response | No correlated response | % of correlated responses that are positive |
| --- | --- | --- | --- | --- |
| NaCl #1 | 7 | 2 | 0 | 77.7778 |
| DMSO | 7 | 1 | 0 | 87.5 |
| Bleach | 9 | 0 | 0 | 100 |
| CA | 9 | 0 | 1 | 90 |
| CA no gluc. | 7 | 2 | 1 | 70 |
| EtOH no gluc. | 5 | 4 | 1 | 50 |
| NaOH (0.05M) | 2 | 6 | 2 | 20 |
| NaOH (0.04M) | 2 | 4 | 3 | 22.22222 |
| EtOH | 6 | 2 | 2 | 60 |
| NaCl #2 | 9 | 0 | 1 | 90 |
| Glycerol | 8 | 1 | 1 | 80 |
| Acetate | 8 | 2 | 0 | 80 |
| CaN0_3_ | 5 | 2 | 0 | 71.42857 |
| total | 84 | 28 | 10 | 68.85246 |

Growth rate

| Evolution Environment | Positive correlated response | Negative Correlated response | No correlated response | % of correlated responses that are positive |
| --- | --- | --- | --- | --- |
| NaCl #1 | 6 | 2 | 1 | 66.6666667 |
| DMSO | 7 | 0 | 1 | 87.5 |
| Bleach | 8 | 1 | 0 | 88.8888889 |
| CA | 5 | 4 | 1 | 50 |
| CA no gluc. | 7 | 3 | 0 | 70 |
| EtOH no gluc. | 1 | 6 | 3 | 10 |
| NaOH (0.05M) | 1 | 8 | 1 | 10 |
| NaOH (0.04M) | 3 | 6 | 0 | 33.3333333 |
| EtOH | 3 | 7 | 0 | 30 |
| NaCl #2 | 9 | 1 | 0 | 90 |
| Glycerol | 6 | 2 | 2 | 60 |
| Acetate | 7 | 2 | 1 | 70 |
| CaN0_3_ | 2 | 4 | 1 | 28.5714286 |
| total | 65 | 46 | 11 | 53.2786885 |

Max OD

| Evolution Environment | Positive correlated response | Negative Correlated response | No correlated response | % of correlated responses that are positive |
| --- | --- | --- | --- | --- |
| NaCl #1 | 6 | 2 | 1 | 66.6666667 |
| DMSO | 7 | 0 | 1 | 87.5 |
| Bleach | 9 | 0 | 0 | 100 |
| CA | 8 | 1 | 1 | 80 |
| CA no gluc. | 6 | 3 | 1 | 60 |
| EtOH no gluc. | 5 | 4 | 1 | 50 |
| NaOH (0.05M) | 2 | 8 | 0 | 20 |
| NaOH (0.04M) | 3 | 6 | 0 | 33.3333333 |
| EtOH | 5 | 4 | 1 | 50 |
| NaCl #2 | 10 | 0 | 0 | 100 |
| Glycerol | 7 | 2 | 0 | 77.7777778 |
| Acetate | 8 | 2 | 0 | 80 |
| CaN0_3_ | 4 | 2 | 1 | 57.1428571 |
| total | 80 | 34 | 7 | 66.1157025 |

Composite (Max OD x growth rate)

Supplemental Figure 1. The number of positive and negative correlated responses are shown for each evolved genotype and each fitness metric. If the difference between the ancestor and evolved genotype was less than 5% we counted it as no correlated response.
